# Four new species of isopods (Crustacea, Isopoda) from South Korea

**DOI:** 10.3897/zookeys.1010.59101

**Published:** 2021-01-13

**Authors:** Sung Hoon Kim, Seong Myeong Yoon

**Affiliations:** 1 Research Center for Endangered Species, National Institute of Ecology, Yeongyang, 36531, South Korea; 2 Department of Biology, College of Natural Sciences, Chosun University, Gwangju 61452, South Korea; 3 Educational Research Group for Age-associated Disorder Control Technology, Graduate School, Chosun University, Gwangju 61452, South Korea

**Keywords:** *
Amakusanthura
*, *
Apanthura
*, *
Idarcturus
*, Korea, *
Neastacilla
*, new species, sublittoral zone

## Abstract

Four new isopods, *Amakusanthura intermedia***sp. nov.**, *Apanthura laevipedata***sp. nov.**, *Idarcturus trispinosus***sp. nov.**, and *Neastacilla paralongipectus***sp. nov.**, are reported from the sublittoral zones in Korean waters. *Amakusanthura intermedia***sp. nov.** differs from its congeners by the following features: the uropodal exopod is sinuous distally and with pointed apex; the maxillipedal endite is present and reaching to the distal end of fused palp articles I and II; and the propodal palm of pereopod I is stepped. *Apanthura laevipedata***sp. nov.** can be distinguishable from its related species by the following characteristics: the eye is lacking; the propodal palm of pereopod I is not stepped; and the uropodal exopod is not sinuous. *Idarcturus trispinosus***sp. nov.** is diagnosed by the following features: the cephalon has three dorsal spines and a pair of lateral spines; pereonite IV has two pairs of small dorsolateral spines, four pairs of dorsal spines, and one posterior spine; and the pleotelson has three pairs of wings laterally. *Neastacilla paralongipectus***sp. nov.** can be distinguished by the following characteristics: the body is smooth and lacking dorsal spines or tubercles; pereonite IV is approximately 5.4× longer than pereonites II and III together; and the pleotelson has two pairs of lateral wings. In this paper, detailed descriptions and illustrations of the four species are presented. A key to the genera of the family Arcturidae and keys to the species of the four genera are also provided.

## Introduction

The Anthuridae Leach, 1814, characterized by having an elongate cylindrical body and the exopod of uropod attaching to the protopod dorsally, is a large family of marine isopods that dominated in the tropical regions (Poore 2001a; Poore and Bruce 2012; Chew et al 2014). Anthurids usually live in sediment burrows as abundant and important components of the offshore soft-sediment marine environment, also commonly occurring in algal mats on hard substrates in both littoral and sublittoral habitats (Brusca and Iverson 1985). Among the 26 anthurid genera, *Amakusanthura* Nunomura, 1977 and *Apanthura* Stebbing, 1900 each contain 43 species and are the largest genera in the family (Boyko et al. 2008a, b). It is known that the *Amakusanthura* is more common in the tropical regions and the *Apanthura* is more abundant in the temperate regions (Poore 2001a). In the Far East of the temperate region, four *Amakusanthura* species have been reported only from Japan (Nunomura 2016) and six *Apanthura* species from Russia (the Kuril Islands), Japan, and Korea (Mezhov 1976; Wägele 1984; Nunomura 1993; Song and Min 2015).

The Arcturidae Dana, 1849 is another large family of marine isopods that dominated in the Arctic region (Poore and Bruce 2012). Arcturids inhabit the sea floor from the subtidal region to the deep sea, feeding by filtration using setose pereopods I–IV (King 2003a; Castelló et al. 2016). Among the 14 arcturid genera, the *Idarcturus* Barnard, 1914 is a small genus comprising only three species with limited distribution: *I. platysoma* Barnard, 1914, from Cape Town, South Africa (Barnard 1914); *I. hedgpethi* Menzies, 1951 and *I. allelomorphus* Menzies & Barnard, 1959, both from California, USA (Menzies 1951; Menzies and Barnard 1959). On the other hand, the *Neastacilla* Tattersall, 1921 comprises 49 species reported worldwide, including 18 species from the Far Eastern Russia and Japan (Richardson 1909; Gurjanova 1936; Kussakin 1971, 1974, 1982; Kussakin and Vasina 1990; Nunomura 2004, 2006, 2008; Boyko et al. 2008c; Golovan et al. 2018). The *Neastacilla* has not yet been reported from Korea.

In this study, we describe two new anthurids, *Amakusanthura intermedia* sp. nov. and *Apanthura laevipedata* sp. nov., and two new arcturids, *Idarcturus trispinosus* sp. nov. and *Neastacilla paralongipectus* sp. nov., from the sublittoral zones in Korean waters. We also provide a key to the genera of the family Arcturidae and keys to the species of these four genera. This is the first reports of *Amakusanthura*, *Idarcturus*, and *Neastacilla* from Korea.

## Materials and methods

**Figure 1.**
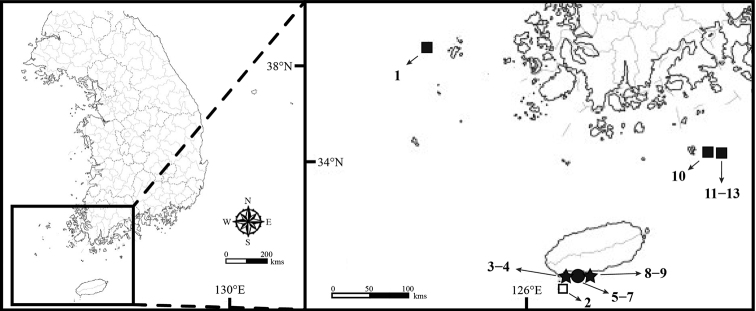
Map of the sampling stations where the isopod specimens collected (station numbers and localities are listed in Table 1). Key: □ *Amakusanthura intermedia* sp. nov. ★ *Apanthura laevipedata* sp. nov. ■ *Idarcturus trispinosus* sp. nov. ● *Neastacilla paralongipectus* sp. nov.

The materials of the present study were collected from 13 sampling stations of the sublittoral zones in Korean waters by SCUBA diving and using a Smith-McIntyre grab (Fig. 1; Table 1). The collected materials were sorted using a sieve with a 1 mm mesh and immediately fixed in 94% ethyl alcohol. The observation and dissection of materials were conducted under a dissecting microscope (Nikon SMZ 1500) and a compound microscope (Olympus BX 50). Measurements and drawings of the specimens were performed with the aid of a drawing tube. The drawings were scanned, inked, and arranged digitally using the methods described by Coleman (2003, 2009). The examined materials in this study were deposited at the National Institute of Biological Resource (**NIBR**) and Chosun University in Korea (**CUK**).

**Table 1.** Sampling stations of the marine isopods in Korean waters.

No.	Locality	Geographical coordinates	Depth (m)	Collecting method	Date of collection
1	Jeollanam-do, Sinan-gun, Heyksan-myeon, Hondo-ri, Hongdo Island	34°40'09"N, 125°10'59"E	10 m	SCUBA diving	19 Jun 2018
2	Jeju-do, Seogwipo-si, Daejeong-eup, Gapa-ri	33°06'54"N, 126°16'42"E	71 m	Smith-McIntyre grab	31 Jan 2018
3	Jeju-do, Seogwipo-si	33°13'00"N, 126°19'30"E	30 m		31 Jan 2018
4		33°11'24"N, 126°18'18"E	30 m		28 Apr 2018
5	Jeju-do, Seogwipo-si, Beophwan-dong	33°13'36"N, 126°32'12"E	33 m		01 Feb 2018
					26 Apr 2018
6	Jeju-do, Seogwipo-si, Seohong-dong	33°13'48"N, 126°33'06"E	51 m	Smith-McIntyre grab	26 Apr 2018
7	Jeju-do, Seogwipo-si, Donghong-dong	33°13'48"N, 126°34'36"E	59 m		26 Apr 2018
8	Jeju-do, Seogwipo-si	33°13'12"N, 126°32'12"E	33 m		01 Feb 2018
9		33°13'54"N, 126°36'24"E	38 m		26 Apr 2018
10	Jeollanam-do, Yeosu-si, Samsan-myeon, Geomun-ri, Sosambudo Island	34°02'23"N, 127°21'43"E	15 m	SCUBA diving	27 Jun 2017
11		34°03'04"N, 127°35'13"E	15 m		28 Jun 2017
12		34°03'15"N, 127°35'00"E	15 m		28 Jun 2017
13		34°03'38"N, 127°35'01"E	15 m		28 Jun 2017

## Taxonomy

### Order Isopoda Latreille, 1817

**Suborder Cymothoida Wägele, 1989**

**Family Anthuridae Leach, 1814**

### Genus *Amakusanthura* Nunomura, 1977

#### Amakusanthura intermedia sp. nov.

Taxon classificationAnimaliaIsopodaAnthuridae

1192C32B-5CB6-537A-BF7F-A88A37CA69E7

http://zoobank.org/740A3A8C-3C00-4E4B-AC7D-409FA2A8A7F2

Figures 2 , 3 , 4

##### Material examined.

***Holotype*.** South Korea • 1 non-ovigerous ♀ (5.4 mm); Jeju-do, Seogwipo-si, Daejeong-eup, Gapa-ri; 33°06'54"N, 126°16'42"E; 71 m; 31 Jan. 2018; Smith-McIntyre grab; NIBRIV0000862806.

***Paratypes*.** 2 ♀♀, same data as for holotype; NIBRIV0000880420.

##### Etymology.

The specific name, *intermedia*, originates from the Latin word *intermedius*, meaning “that is between”. This name refers to the length of maxillipedal endite comparing to the related species.

##### Description of holotype female.

**Figure 2.**
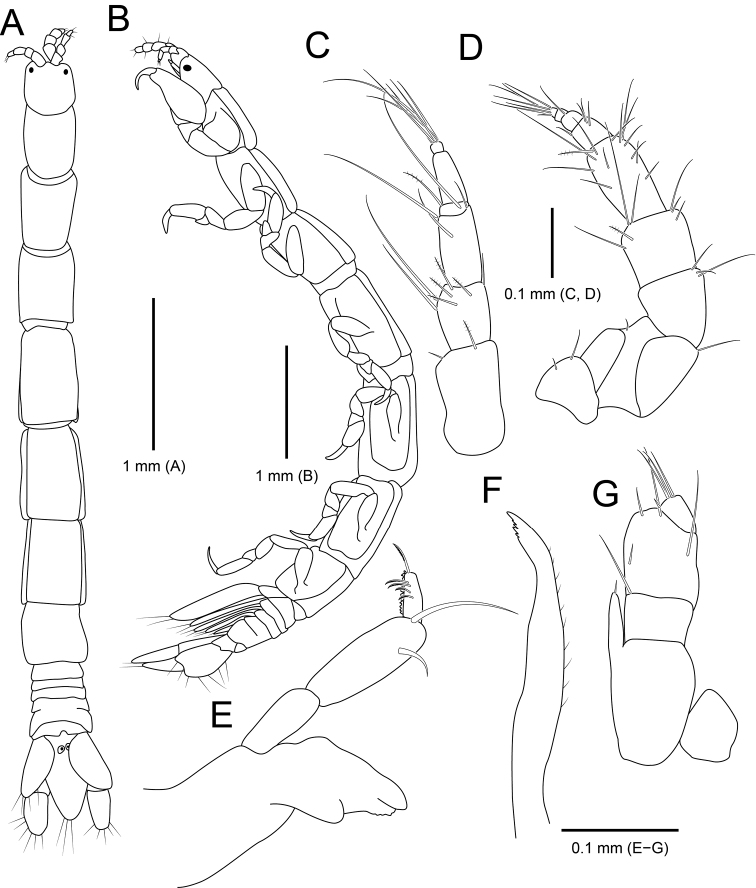
*Amakusanthura intermedia* sp. nov., holotype, female **A** habitus, dorsal view **B** habitus, lateral view **C** antennule **D** antenna **E** mandible **F** maxilla **G** maxilliped.

*Body* (Fig. 2A, B) 5.4 mm, 13× longer than wide, smooth and slender. Cephalon square to globular, 0.8× as long as pereonite I; rostrum as long as anterolateral lobes; eye very small; color not pigmented, white. Pereonites rectangular; pereonites I–VI similar to each other in length; pereonite VII ~ 0.7× as long as pereonite VI; coxal plates of pereonites IV–VI visible dorsally. Pleonites 1.2× longer than pereonite VII; pleonites I–V separated by folds except dorsally between pleonites IV and V; pleonites IV and V visible partial sutures laterally; pleonite VI visible dorsally, with dorsal notch posteriorly.

*Antennule* (Fig. 2C) composed of three peduncular and three flagellar articles, sequentially slender distally in width. Peduncular article I rectangular, with one simple and a single penicillate seta distally; article II oblong, 0.6× as long as article I, with three simple and four penicillate setae distally; article III rectangular, 1.2× longer than article II, with three simple setae distally. Flagellar article I 0.4× as long as wide, with one simple and one penicillate seta; article II elongated, ~ 4× longer than article I, without seta; article III minute, square, with seven simple setae distally.

*Antenna* (Fig. 2D) consisting of five peduncular and four flagellar articles. Peduncular article I ~ 0.4× as long as article II, with two simple setae laterally; article II 1.8× longer than article I, with one short simple seta laterally and one simple seta distally; article III 0.7× as long as article II, with three simple setae distally; article IV square, subequal to article III, with six simple setae and one penicillate seta distally; article V elongate, 1.4× longer than article IV, with 13 simple setae on both lateral margin and one penicillate seta on distal end. Flagellar articles much shorter than peduncular article V, consecutively shortened; article I with four simple setae distally; article II with one simple seta; article III without setae; article IV with seven simple setae; articles III and IV minute.

*Mandible* (Fig. 2E), incisor with one prominent cusp and serrated margin; molar process bluntly rounded; palp article II 1.5× longer than article I, slightly stouter than other articles, with two simple setae distally; article III shortest in length, with four serrated setae and a row of spines laterally, one serrated seta distally.

*Maxilla* (Fig. 2F) with five teeth distally and several fine setae laterally.

*Maxilliped* (Fig. 2G), endite extending to proximal region of palp article III, with one short simple seta apically; palp articles I and II fused, with one simple seta distally; article III free, with two simple setae laterally and two simple setae distally; articles IV and V fused, oblique, smaller than other articles, with four simple setae laterally.

**Figure 3.**
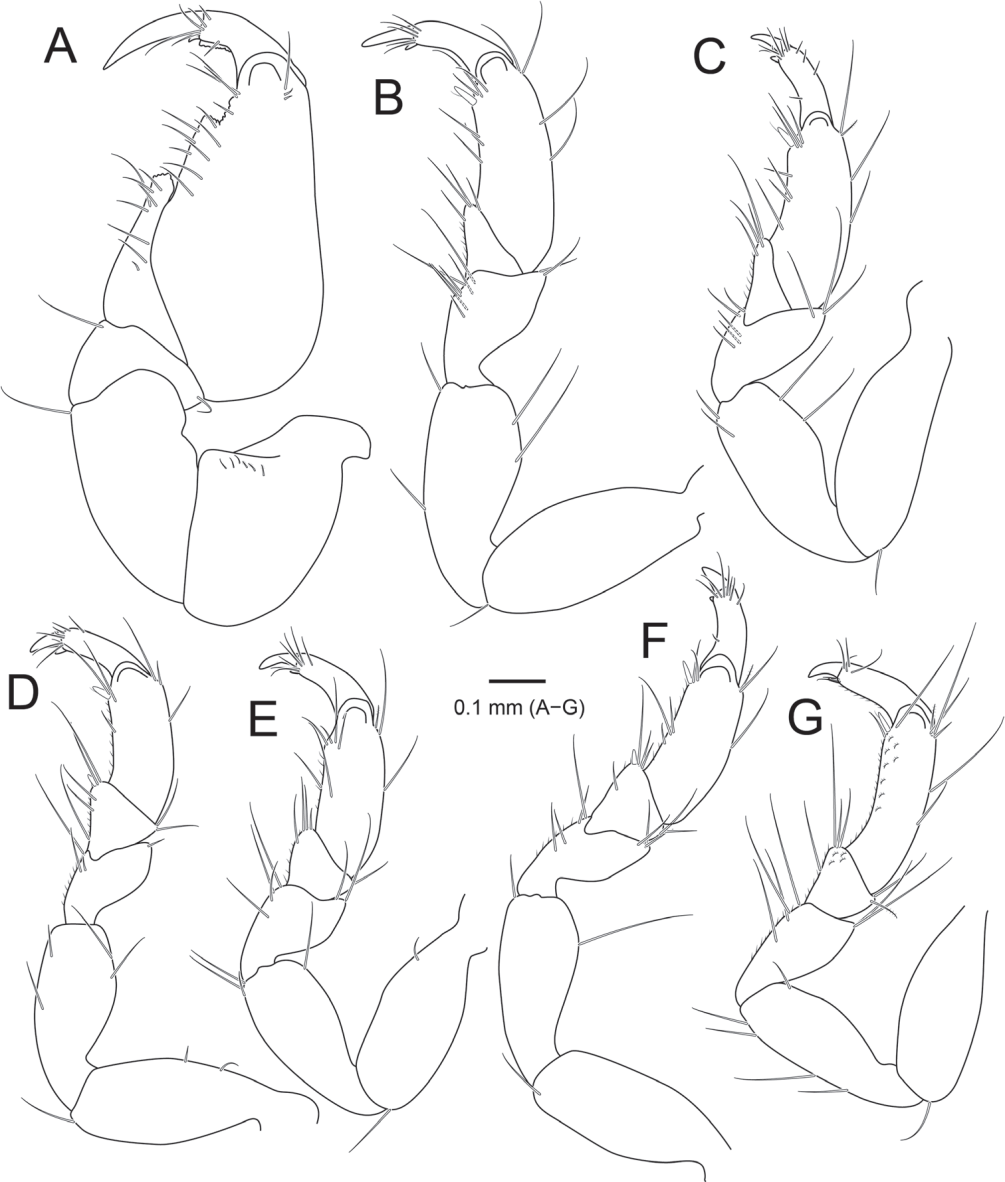
*Amakusanthura intermedia* sp. nov., holotype, female **A** pereopod 1 **B** pereopod 2 **C** pereopod 3 **D** pereopod 4 **E** pereopod 5 **F** pereopod 6 **G** pereopod 7.

*Pereopod I* (Fig. 3A), basis continuously stouter distally, with three simple setae and three penicillate setae on superior margin; ischium slender than basis, rectangular, with one simple seta on inferodistal end; merus much shorter than ischium, wider than long, with one simple seta on superior and inferior distal end, respectively; carpus triangular, protruding inferodistally, with rough margin inferodistally and eight simple setae along with inferior margin; propodus stepped on palm, with twelve simple setae on inferior margin and three simple setae on distal end; dactylus with five simple setae distally, a row of spines and one simple seta laterally; unguis as long as dactylus, much longer than those of other pereopods, with small accessory unguis. *Pereopods II and III* (Fig. 3B, C), basis elongate, oval, with one simple seta on inferodistally; ischium slightly shorter than basis, with two simple setae on both lateral margins; merus ~ 0.5× as long as ischium, tapering proximally, with several simple setae on superior and inferior margins; carpus triangular, with several simple setae on inferodistal angle and fine setae along inferior margin; propodus elongate and oval, with simple setae on both lateral margins and one stout seta on inferodistal angle; dactylus with simple setae distally; unguis 0.5× as long as dactylus, with small accessory unguis. *Pereopods IV-VI* (Fig. 3D–F), carpus more or less rectangular, with one stout seta on inferodistal angle (absent in pereopod V); propodus with several fine setae along both lateral margins and one stout seta on inferodistal angle. *Pereopod VII* (Fig. 3G), carpus and propodus with dentate margin baring simple setae along inferior margin.

**Figure 4.**
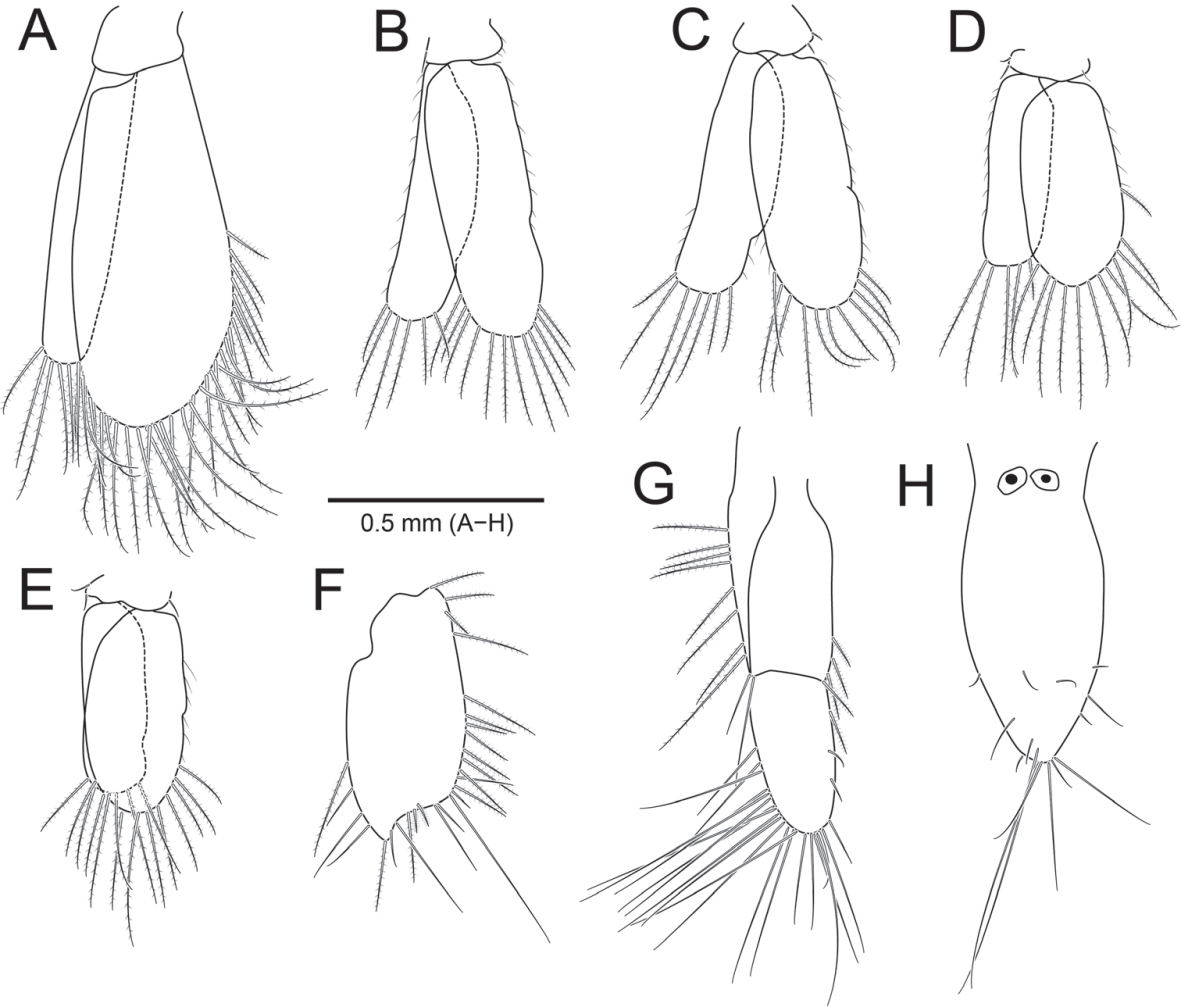
*Amakusanthura intermedia* sp. nov., holotype, female **A** pleopod 1 **B** pleopod 2 **C** pleopod 3 **D** pleopod 4 **E** pleopod 5 **F** uropodal exopod **G** uropodal endopod **H** pleotelson.

*Pleopods* (Fig. 4A–E), exopod slightly longer than endopod. *Pleopod I* (Fig. 4A), exopod ~ 2× as long as wide; endopod 0.4× wider than exopod. *Pleopods II–V* (Fig. 4B–E), protopod with one or two simple setae on inner or outer margin.

*Uropod* (Fig. 4F, G), protopod rectangular, 2.4× longer than wide, with plumose setae both lateral margins; exopod oval, almost 2× longer than wide, surrounded by plumose and simple setae, with pointed apex, sinuous distally; endopod with plumose and simple setae along both lateral margins.

*Pleotelson* (Fig. 4H) 2.2× longer than wide, concave proximally, convex medially, tapering distally, with two statocysts on dorsal surface proximally; apex with five simple setae; distal region with several simple setae on dorsal surface and lateral margins.

##### Distribution.

Southern coast of Jeju-do in South Korea.

##### Habitat.

Sublittoral zone of sandy bottom.

##### Remarks.

*Amakusanthura intermedia* sp. nov. is most similar to four species, *A. magnifica* (Menzies & Frankenberg, 1966), *A. pimelia* (Poore & Lew Ton, 1985), *A. paramagnifica* Müller, 1992, and *A. tengo* Müller, 1992, in having the following characteristics: (1) pleonites I–III are visible by folds dorsally and laterally, while pleonites IV and V are visible laterally; (2) the uropodal exopod has sinuous and acute distal region; (3) the pleotelson is concave laterally and tapering distally; (4) the maxillipedal endite is present; and (5) the propodal palm of pereopod I is stepped (Schultz 1969; Poore and Lew Ton 1985; Kensley and Schotte 1989; Müller 1992). However, the new species can be distinguished from the latter species by having the maxillipedal endite (vs. lacking in *A. tengo*), the carpus of pereopod I protruding inferodistally (vs. not protruding in *A. pimelia*), the maxillipedal endite reaching to the distal end of fused articles I and II (vs. over in *A. magnifica* and not reaching in *A. paramagnifica*) (Schultz 1969; Poore and Lew Ton 1985; Kensley and Schotte 1989; Müller 1992).

#### Key to known species of the genus *Amakusanthura* in the Far East

**Table:** 

1	Pleonites IV and V distinguished by dorsal fold	***A. aokii* Nunomura, 2004**
–	Pleonites IV and V not distinguished by dorsal fold	**2**
2	Pleonites I–III not indicated by dorsal folds	**3**
–	Pleonites I–III indicated by dorsal folds	**4**
3	All pereonites without dorsal pits	***A. azumai* Nunomura, 2016**
–	Pereonites IV–VII with dorsal pits	***A. longiantennata* Nunomura, 1977**
4	Propodal palm of pereopod I not stepped	***A. aokii* Nunomura, 2004**
–	Propodal palm of pereopod I stepped	***A. intermedia* sp. nov.**

### Genus *Apanthura* Stebbing, 1900

#### Apanthura laevipedata sp. nov.

Taxon classificationAnimaliaIsopodaAnthuridae

0DA03D10-9ACD-515F-855E-72C92EFD8A9D

http://zoobank.org/748471B2-B1F1-4E58-8AB1-72F3D81164FC

Figures 5 , 6 , 7

##### Material examined.

***Holotype*.** South Korea • 1 non-ovigerous ♀ (5.5 mm); Jeju-do, Seogwipo-si, Beophwan-dong; 33°13'36"N, 126°32'12"E; 33 m; 26 Apr. 2018; Smith-McIntyre grab; NIBRIV0000862805.

***Paratypes*.** 4 ♀♀, same data as for holotype; 2 ♀♀, same locality as for holotype; 1 Feb. 2018; 2 ♀♀; Jeju-do, Seogwipo-si, Seohong-dong; 33°13'48"N, 126°33'06"E; 51 m; 26 Apr. 2018; Smith-McIntyre grab • 1 juvenile ♀; Jeju-do, Seogwipo-si, Donghong-dong; 33°13'48"N, 126°34'36"E; 59 m; 26 Apr. 2018; Smith-McIntyre grab; NIBRIV0000880421.

##### Etymology.

The specific name, *laevipedata*, originates from the combination of the Latin words *laevis*, meaning smooth and *pedis*, meaning foot. This name refers to having the propodus of pereopod that is smooth, not stepped.

##### Description of holotype female.

**Figure 5.**
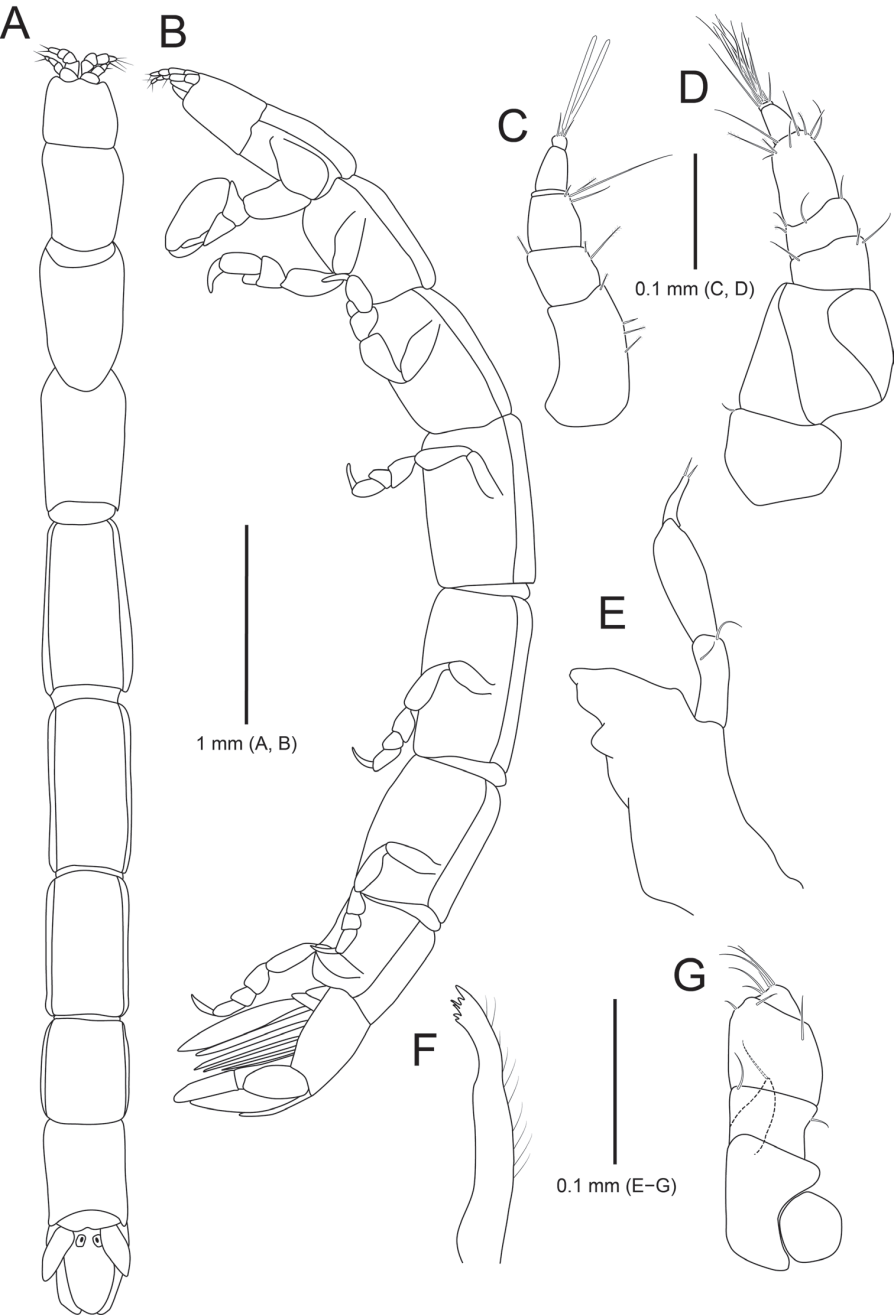
*Apanthura laevipedata* sp. nov., holotype, female **A** habitus, dorsal view **B** habitus, lateral view **C** antennule **D** antenna **E** mandible **F** maxilla **G** maxilliped.

*Body* (Fig. 5A, B) smooth and slender, 5.5 mm in length, 11× longer than wide. Cephalon square, 0.7× as long as pereonite I; rostrum extending as long as anterolateral lobes; eye absent. Pereonites oblong; pereonites I–III similar in length; pereonites IV–VI longer than previous three pereonites, subequal each other in length; pereonite VII ~ 0.7× as long as pereonite VI; coxal plates of pereonites V–VII visible dorsally. Pleonites I–V not separated by folds dorsally and laterally; pleonite VI with middorsal notch posteriorly.

*Antennule* (Fig. 5C) consisting of three peduncular and three flagellar articles. Peduncular article I oblong, longer than wide, with three penicillate setae and one simple seta along lateral margin; article II square, ~ 0.5× as long as article I, with three penicillate setae on distal end; article III similar to article II in length, slender than article II, with one penicillate seta and three simple setae distally. Flagellar article I much shorter than other articles, 0.1× as long as peduncular article III; article II oblong to oval, tapering distally, 0.8× as long as peduncular article III; article III square to globular, 0.3× as long as article II, with two simple setae and two aesthetascs distally.

*Antenna* (Fig. 5D) longer than antennule, composed of five peduncular and three flagellar articles. Peduncular article I rectangular to globular, with one simple seta distally; article II 1.5× longer than article I; article III oblong, 0.4× as long as article II, with three simple setae distally; article IV similar to article III in shape, 0.7× as long as article III, with four simple setae distally; article V elongated oblong, 2.3× longer than article IV, with three penicillate and four simple setae on distal end. Flagellar article I elongated rectangular, ~ 0.5× as long as peduncular article V; articles II and III minute, with several simple setae on distal end.

*Mandible* (Fig. 5E), incisor with one cusp; molar process blunt; palp article I elongated oblong, with two simple setae distally; article II 1.4× longer than article I, slightly thicker than other articles; article III slander, slightly tapering distally, 0.4× as long as article II, with two short simple setae distally.

*Maxilla* (Fig. 5F) with six irregular teeth distally and fine setae laterally.

*Maxilliped* (Fig. 5G), epipods globular; endite slightly exceeding distal end of fused palp articles I and II, tapering distally, with one simple seta distally; palp articles I and II fused, rectangular, with one simple seta distally and one simple seta laterally; article III free, almost square, with three simple setae on distal end; articles IV and V fused, triangular, much smaller than other palp articles, with five simple setae along inner margin.

**Figure 6.**
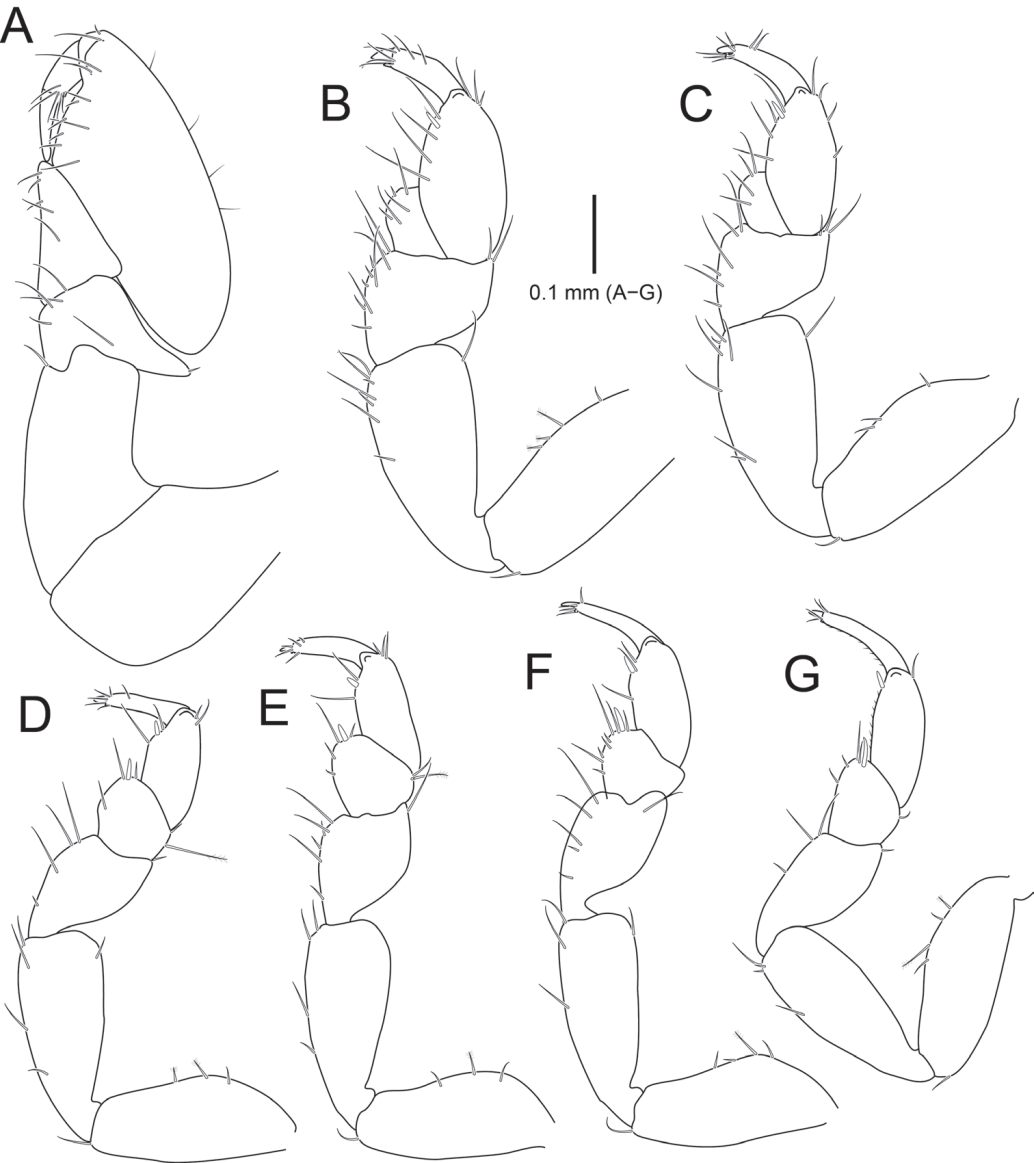
*Apanthura laevipedata* sp. nov., holotype, female **A** pereopod 1 **B** pereopod 2 **C** pereopod 3 **D** pereopod 4 **E** pereopod 5 **F** pereopod 6 **G** pereopod 7.

*Pereopod I* (Fig. 6A) basis as long as ischium, gradually tapering proximally; ischium rectangular, with one simple seta on inferodistal angle; merus 0.4× as long as ischium; superior margin of merus extending upwardly, with five simple setae on inferior margin, one short simple seta on superior margin, and one simple seta on mesial margin; carpus triangular, with five simple setae along inferior margin; propodus not stepped on palm, with two rows of simple setae along inferior margin and several fine setae on superior margin; dactylus oblique, with five simple setae; unguis slightly longer than dactylus; much longer than other pereopods, with accessory unguis distally. *Pereopods II*–*VII* (Fig. 6B–G) basis oblong to oval, with several simple setae and penicillate setae on superior margin and one simple seta on inferodistal angle; ischium subequal in length, gradually tapering proximally, with several simple setae along with inferior margin and none or one simple seta on superodistal angle; merus tapering proximally, with several simple setae on inferior margin and 1–3 simple setae on superior margin; carpus of pereopods II and III triangular, with several simple setae on inferior margin without stout seta on inferodistal angle; carpus of pereopods IV–VII subsquare to globular or trapezoidal, with one stout seta on inferodistal angle, several simple setae on both lateral margins, none or one penicillate seta on superior margin; propodus slightly elongate and oval, with several simple setae on both lateral margins and one stout seta on inferodistal angle; dactylus slender, with several simple setae distally, unguis, much shorter than dactylus, with minute accessory unguis on distal end.

**Figure 7.**
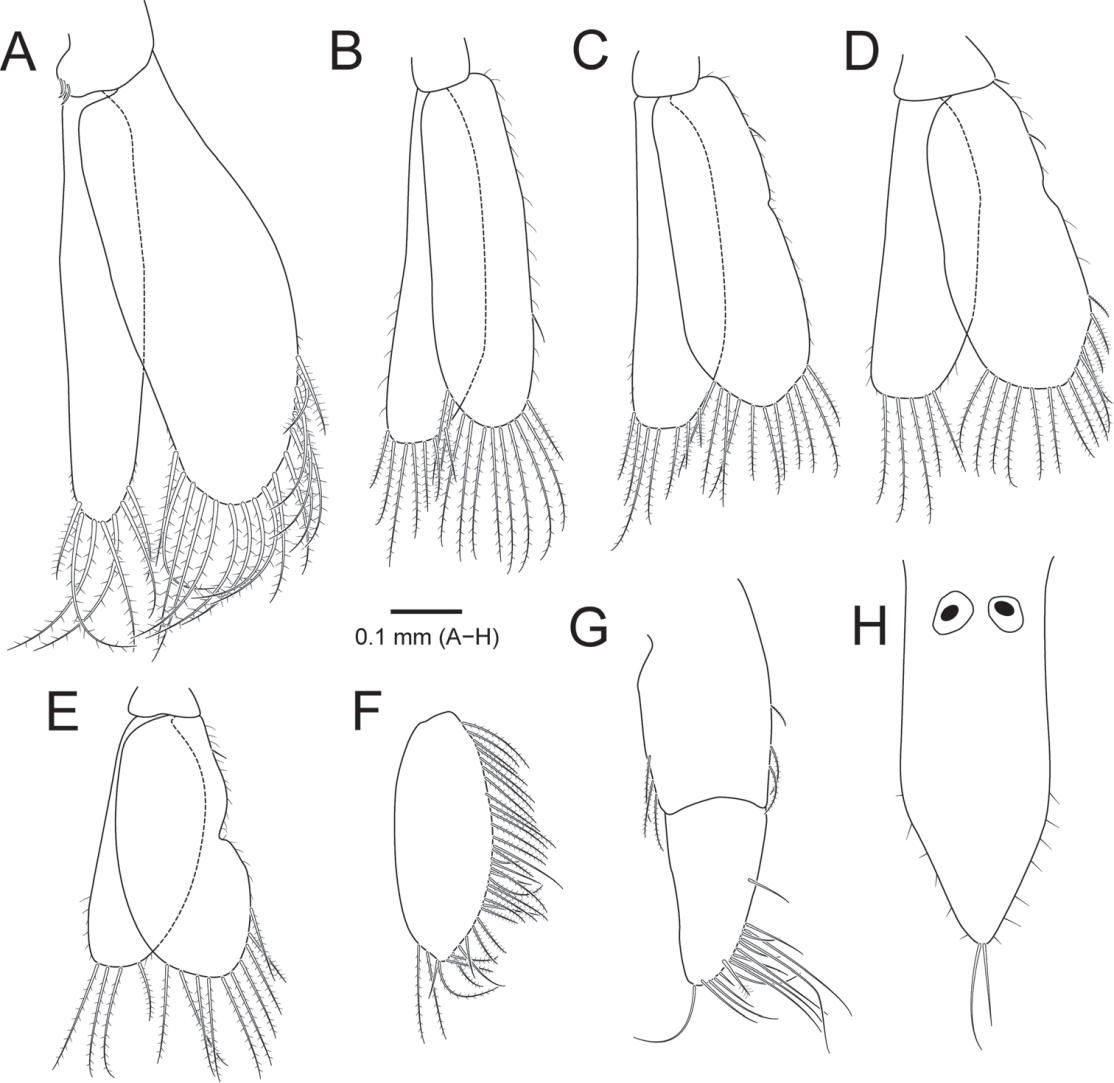
*Apanthura laevipedata* sp. nov., holotype, female **A** pleopod 1 **B** pleopod 2 **C** pleopod 3 **D** pleopod 4 **E** pleopod 5 **F** uropodal exopod **G** uropodal endopod **H** pleotelson.

*Pleopods* (Fig. 7A–E), protopod square to rectangular; rami subequal in length, rounded distally. *Pleopod I* (Fig. 7A), protopod with three coupling hooks on inner margin; exopod 2× wider than endopod. *Pleopods II–V* (Fig. 7B–E), exopod with one simple seta on outer margin; pleopods III–V with notch on outer margin.

*Uropod* (Fig. 7F, G), protopod oblong, 1.7× longer than wide, with three plumose setae on each lateral margin; endopod triangular to oval, with one penicillate seta and 16 simple setae along outer margin; exopod oval, with numerous plumose and several simple setae along outer margin, not sinuous distally.

*Pleotelson* (Fig. 7H) 2.6× longer than wide, with two statocysts dorsally; lateral margins parallel; apex rounded, tapering distally, with two simple setae distally.

##### Distribution.

Southern coast of Jeju-do in South Korea.

##### Habitat.

Sublittoral zone of sandy bottom.

##### Remarks.

*Apanthura laevipedata* sp. nov. differs from its congeners by the following features combined: (1) the integument is not pigmented; (2) the eyes are lacking; (3) pleonites I–V are not separated by folds dorsally and laterally; (4) the propodal palm of pereopod I is not stepped; and (5) the uropodal exopod is not sinuous.

Besides the new species, four species that have no eyes are known in the genus *Apanthura* Stebbing, 1900: *A. insignifica* Kensley, 1978; *A. tyrrhenica* Wägele, 1980; *A. drosera* Poore & Lew Ton, 1985; and *A. fusei* Nunomura, 1993 (Kensley 1978; Wägele 1980; Poore and Lew Ton 1985; Nunomura 1993). Among these species, *Apanthura laevipedata* sp. nov. is most similar to *A. fusei* in terms of dorsally fused pleonites I–V and not sinuous uropodal exopods. However, the new species is easily distinguished from the latter by not stepped propodal palm of pereopod I (vs. stepped in the latter), upwardly extending carpus (vs. not extending in the latter), laterally invisible suture in pleonites I–V (vs. visible in the latter), and parallel lateral margins of the pleotelson (vs. proximally concave lateral margins in the latter) (Nunomura 1993). The new species also differs from *A. drosera* in having not stepped propodal palm of pereopod I (vs. stepped in the latter) (Poore and Lew Ton 1985), while can be distinguished from *A. insignifica* and *A. tyrrhenica* in that all pleonites are not separated by folds (vs. separated in the latter species) (Kensley 1978; Wägele 1980).

#### Key to known species of the genus *Apanthura* in the Far East

**Table:** 

1	Eye present	**2**
–	Eye absent	**6**
2	Propodal palm of pereopod I stepped	***A. honshuensis* Wägele, 1984**
–	Propodal palm of pereopod I not stepped	**3**
3	Statocysts absent	***A. shikokuensis* Nunomura, 1993**
–	Statocysts present	**4**
4	Uropodal exopod not sinuous distally	***A. trioculata* Nunomura, 1993**
–	Uropodal exopod sinuous distally	**5**
5	Distal end of uropodal exopod deeply notched	***A. excavate* Mezhov, 1976**
–	Distal end of uropodal exopod not notched	***A. koreaensis* Song & Min, 2016**
6	Lateral margins of pleotelson concave proximally; propodal palm of pereopod I stepped	***A. fusei* Nunomura, 1993**
–	Lateral margins of pleotelson parallel; propodal palm of pereopod I not stepped	***A. laevipedata* sp. nov.**

### Suborder Valvifera Sars, 1883

#### Family Arcturidae

Taxon classificationAnimaliaIsopodaArcturidae

Dana, 1849

25707C02-CAF4-5016-B6EB-72B0031E4CDF

##### Diagnosis

**(modified after Poore 2001b).** Body moderately cylindrical, geniculate between pereonites IV and V, occasionally straight; cephalon and fused pereonite I expanded ventrolaterally; pereonite IV at least 1.5× longer than pereonite III; pleonites and pleotelson fused. Pereopod I gnathopod-like, immersed in lateral view by lateral plates of cephalon and pereonite I; pereopods II–IV slender, setose, directed anteriorly; pereopods V–VII ambulatory. Pleopod I, protopod elongated, much longer than wide; exopod of male with lateral excavation, and either a tuft of fine setae, long plumose setae, or both. Uropod biramous; endopod much smaller than exopod.

##### Remarks.

Classification of the genera of Arcturidae had difficult because many genera were inadequately and ambiguously described (Kussakin 1972; King 2003b). To resolve this problem, Kussakin (1972) suggested using the structure of pereopods I–IV to diagnose genera and presented a key based on these features to eight Arctic and boreal genera. Since then Poore (2001b) transferred *Antarcturus* zur Strassen, 1903 and related genera Antarcturidae Poore, 2001. *Parapleuroprion* Kussakin, 1972 is now synonym of *Arcturus* Latreille, 1829 (Boyko et al. 2008d). *Arcturella* G. O. Sars, 1897 is synonym of *Astacilla* Cordiner, 1793 (Kensley et al. 2007; Rincón et al. 2018). Here we submit the key to all genera of Arcturidae.

#### Key to genera of the family Arcturidae

**Table:** 

1	Pereopod I with unguis	**2**
–	Pereopod I without unguis	**7**
2	Pereopod IV absent	***Arcturinoides* Kensley, 1977**
–	Pereopod IV present	**3**
3	Pereopods II–IV with flexion between carpus and propodus	**4**
–	Pereopods II–IV without flexion between carpus and propodus	**6**
4	Pereonites without ventral process in both sex	***Astacilla* Cordiner, 1793**
–	Pereonites III or V with ventral process in male	**5**
5	Pereonite III with ventral process in male	***Arcturopsis* Koehler, 1911**
–	Pereonite V with ventral process in male	***Arctopsis* Barnard, 1920**
6	Pereopods II–IV with unguis	***Arcturus* Latreille, 1829**
–	Pereopods II–IV without unguis	***Arcturina* Koehler, 1911**
7	Pereopods III and IV absent	**8**
–	Pereopods III and IV present	**9**
8	Last flagellar article of antenna with rounded tubercle at midlength; maxillipedal palp 5-articled; uropod biramous	***Amesopous* Stebbing, 1905**
–	Last flagellar article of antenna without rounded tubercle at midlength; maxillipedal palp 4-articled; uropod uniramous	***Edwinjoycea* Menzies & Kruczynski, 1983**
9	Pereopod IV vestigial	***Arcturinella* Poisson & Maury, 1931**
–	Pereopod IV not vestigial	**10**
10	Pereopods II–IV with flexion between carpus and propodus	***Agularcturus* Kensley, 1984**
–	Pereopods II–IV without flexion between carpus and propodus	**11**
11	Pereopod II with dactylus, while pereopods III and IV without dactylus	***Parastacilla* Hale, 1924**
–	Pereopods II–IV with dactylus (lacking in few *Neastacilla* species)	**12**
12	Pereopod II with short ungius	***Spectrarcturus* Schultz, 1981**
–	Pereopods II–IV without ungius	**13**
13	Body moderately flattened dorsoventrally; propodus of pereopod I with serrated setae on palmar surface; carpus with serrated setae on ventral margin; exopod of pleopod I with simple setae subbasally in male	***Idarcturus* Barnard, 1914**
–	Body almost cylindrical; propodus of pereopod I without serrated setae on palmar surface; carpus without serrated setae on ventral margin; exopod of pleopod I with plumose setae subbasally	***Neastacilla* Tattersall, 1921**

#### Genus Idarcturus

Taxon classificationAnimaliaIsopodaArcturidae

Barnard, 1914

0ADCB1AF-62BA-5ED9-8A1D-B55889E2D72C

##### Type species.

*Idarcturus platysoma* Barnard, 1914, by monotype.

##### Diagnosis.

Body not or slightly geniculate, moderately flattened dorsoventrally in female, whereas cylindrical in male; pereonite IV longer than other pereonites, but not markedly elongate in both sexes, < 2× longer than pereonites II and III combined. Pereopod I carpus with serrated setae on inferior margin; propodus with serrated setae and comb setae on palmar surface and inferior margin; dactylus with comb setae distally; unguis lacking. Pereopods II–IV lacking flexion between carpus and propodus; dactylus claw-like. Pleopod I, exopod with fine setae subbasally or mesally.

#### Idarcturus trispinosus sp. nov.

Taxon classificationAnimaliaIsopodaArcturidae

8E31490D-95DE-5D0C-804E-FA6EB489CA7E

http://zoobank.org/35DABEAB-C655-4756-805E-6EF4CED60CC5

**Figure 8.**
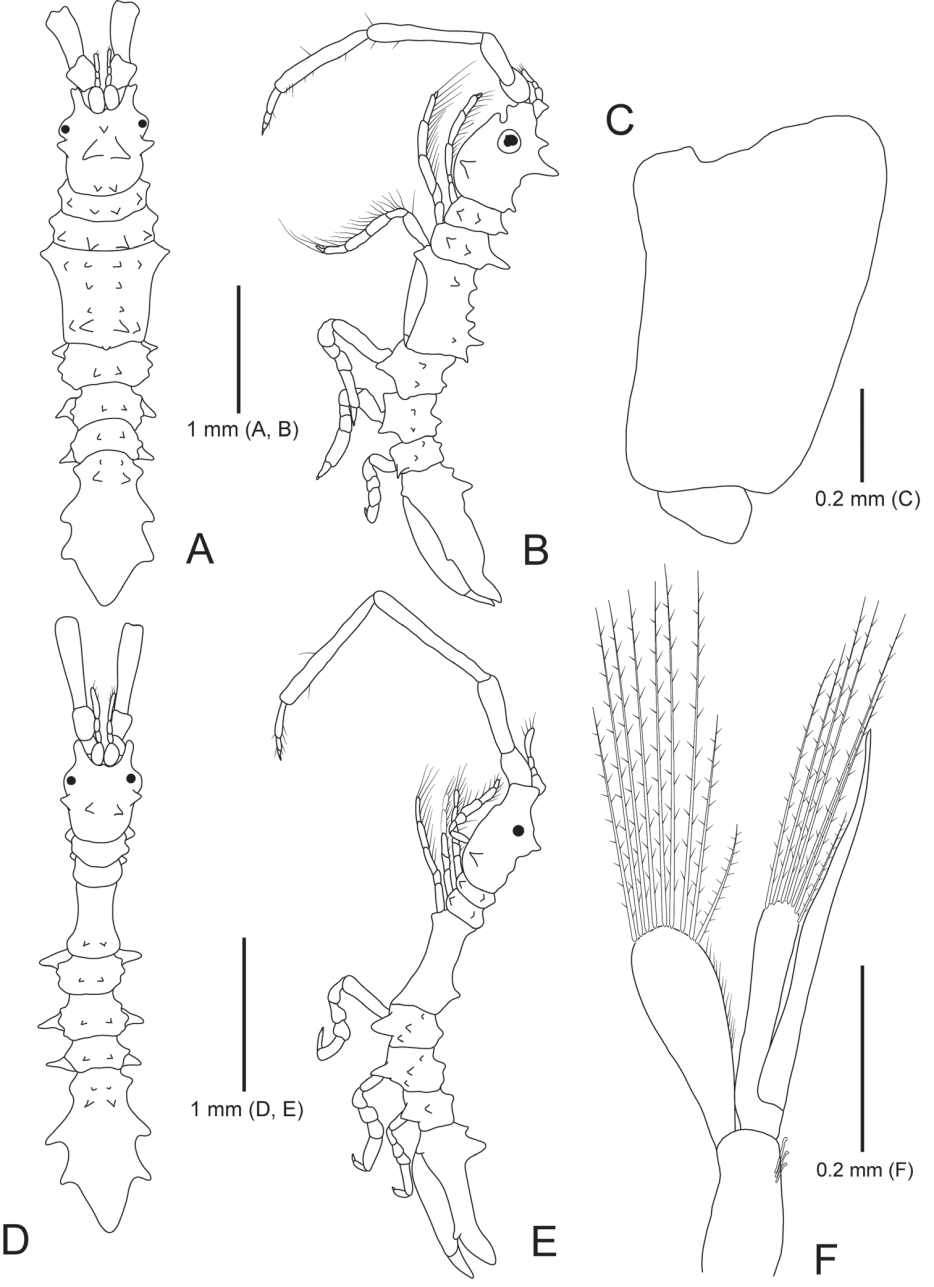
*Idarcturus trispinosus* sp. nov., holotype, female **A** habitus, dorsal view **B** habitus, lateral view **C** oostegite 4. Paratype, male **D** habitus, dorsal view **E** habitus, lateral view **F** pleopod 2.

**Figure 9.**
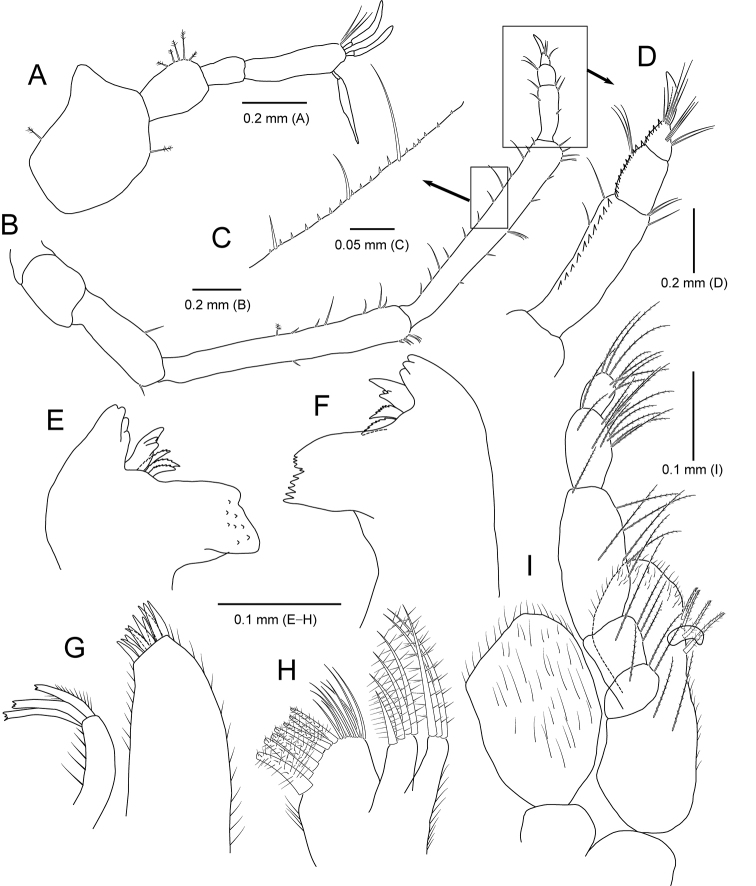
*Idarcturus trispinosus* sp. nov., holotype, female **A** antennule **B** antenna **C** detail of peduncular articles of antenna **D** flagellum of antenna **E** left mandible **F** right mandible **G** maxillule **H** maxilla **I** maxilliped.

**Figure 10.**
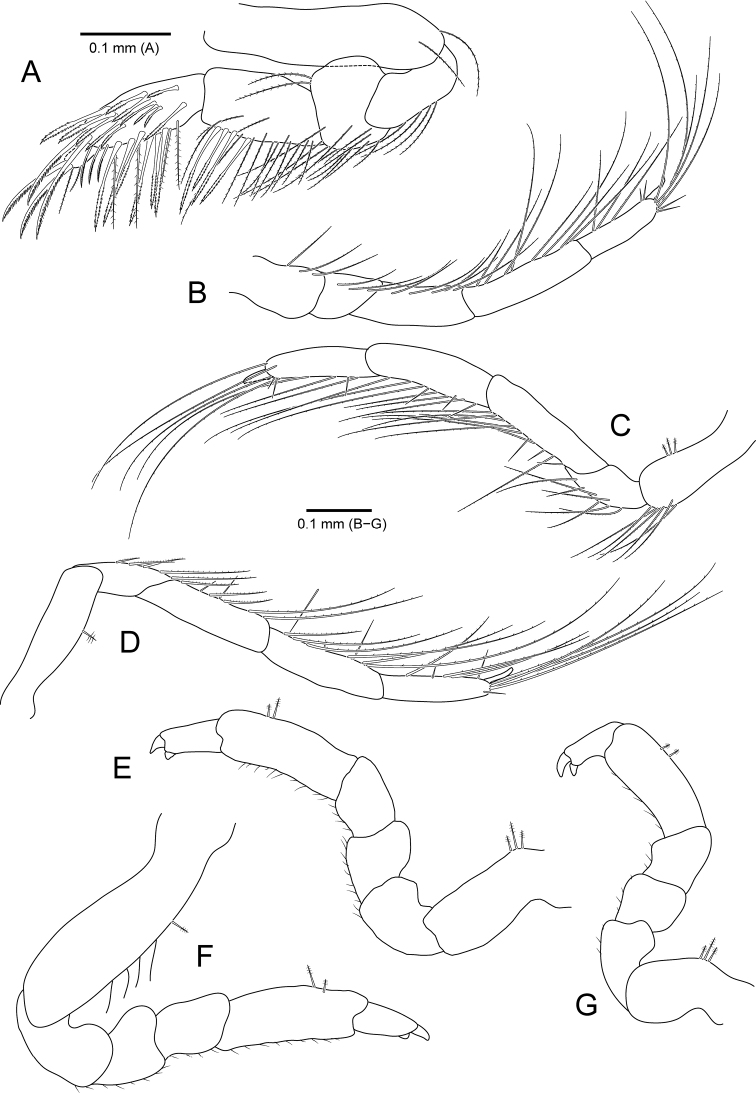
*Idarcturus trispinosus* sp. nov., holotype, female **A** pereopod 1 **B** pereopod 2 **C** pereopod 3 **D** pereopod 4 **E** pereopod 5 **F** pereopod 6 **G** pereopod 7.

**Figure 11.**
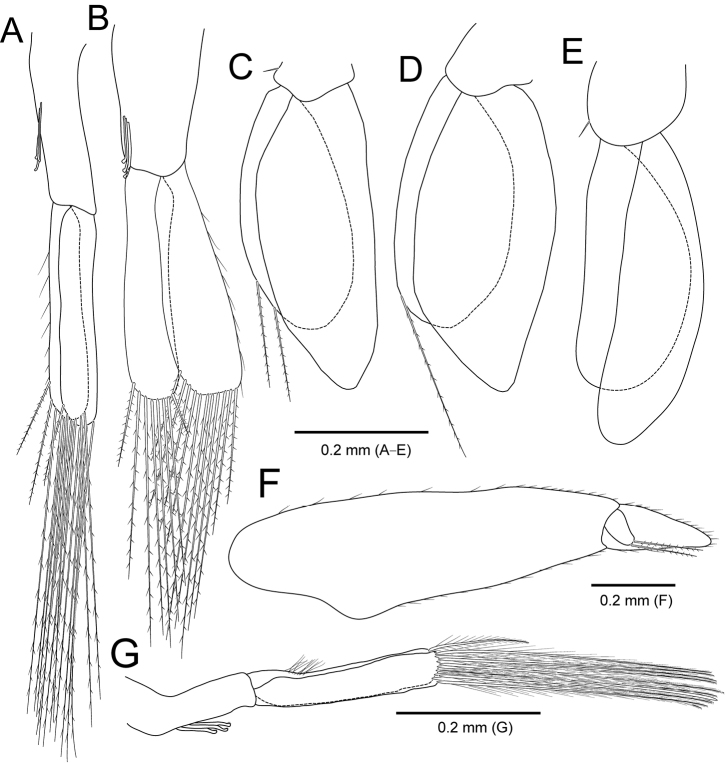
*Idarcturus trispinosus* sp. nov., holotype, female **A** pleopod 1 **B** pleopod 2 **C** pleopod 3 **D** pleopod 4 **E** pleopod 5 **F** uropod. Paratype, male **G** pleopod 1.

Figures 8 , 9 , 10 , 11

##### Material examined.

***Holotype*.** South Korea • 1 ♀ ovigerous (4.5 mm); Jeollanam-do, Yeosu-si, Samsan-myeon, Geomun-ri, Sangbackdo Island; 34°03'4"N, 127°35'13"E; 15 m, 28 Jun. 2017; SCUBA diving; NIBRIV0000813025.

***Paratypes*.** South Korea • 1 ♂ (3.3 mm); Sangbackdo Island; 34°03'15"N, 127°35'00"E; 15 m; 28 Jun. 2017; SCUBA diving • 1 ♀; Sangbackdo Island; 34°03'38"N, 127°35'01"E; 15 m; 28 Jun. 2017; SCUBA diving • 1 ♂, Sosambudo Island; 34°02'23"N, 127°21'43"E; 15 m, 27 Jun. 2017; SCUBA diving; NIBRIV0000880423.

##### Additional material.

South Korea • 1 ♂, 2 ♀♀; Sinan-gun, Heyksan-myeon, Hondo-ri, Hongdo Island, 34°40'09"N, 125°10'59"E; 10 m; 19 Jun. 2018; SCUBA diving.

##### Description of holotype female.

*Body* (Fig. 8A, B) moderately flattened dorsoventrally, slightly geniculated between pereonites IV and V; length 4.5 mm, 4.5× longer than wide. *Cephalon* with three dorsal spines, one pair of lateral spines; anterior margin deeply concave, with small median process; anterolateral lobe expended anteriorly, concave distally; eye large, round, positioned laterally. *Pereonites* with dorsal spines; pereonite I with one pair of dorsal spines; pereonites II and III similar to each other in length, with one pair of dorsal spines, two pairs of lateral spines; dorsal spines of pereonite III bigger than dorsal spines of pereonite II; pereonite IV widest, ~ 1.5× longer than pereonites II and III together, with two pairs of small dorsolateral spines, four pairs of dorsal spines, one middorsal spine on posterior margin; posterior dorsal spines larger than other dorsal spines; anterolateral angle expanded laterally; oostegite IV with suture line posteriorly; pereonites V–VII similar to each other in length, with one pair of small dorsal spines, two or three tuberculate elevations. *Pleon* ~ 1.3× longer than pereonites V–VII, with two pairs of dorsal spines; anterior spines smaller than posterior spines; pleotelson gradually tapering posteriorly, with three pairs of wings laterally; proximal wing smaller than proceeding wings; apex round.

*Antennule* (Fig. 9A) exceeding peduncular article II of antenna, consisting of three peduncular articles and single-articled flagellum; peduncular article I globular, with one projection dorsally and two penicillate setae; article II cylindrical, with five penicillate setae; article III smaller than article II; flagellum with three aesthetascs on anterodistal end and three simple setae on distal end. *Antenna* (Fig. 9B–D) almost half of body length, slender, consisting of five peduncular articles and three flagellar articles; peduncular article I small; article II ×longer than article I; article III ×longer than article II, with two short simple setae distally; articles IV and V similar to each other, almost 3× longer than article III, with numerous minute simple setae along with lateral margin; article IV with one penicillate seta laterally; flagellar article I longer than flagellar articles II and III; articles II and III with one low of spines resembling saw-teeth on ventral margin; article III with one large claw apically.

*Left mandible* (Fig. 9E), incisor weakly 4-toothed; lacinia mobilis 3-toothed, with three serrated setae; molar process broad, rough distally. *Right mandible* (Fig. 9F), incisor 3-toothed; lacinia mobilis 4-toothed, with two serrated setae; molar process broad, strongly serrated. *Maxillule* (Fig. 9G) with fine setae laterally; inner lobe with three trifurcated setae; outer lobe with ten robust setae distally. *Maxilla* (Fig. 9H) with fine setae on lateral margin; inner lobe with seven stout circum-plumose setae on subapical region, nine simple setae on apical margin; mesial lobe with four plumose setae apically; outer lobe with three plumose setae distally. *Maxilliped* (Fig. 9I), palp article I oval, with three plumose setae; article II square, with four plumose setae; article III cylindrical, with seven plumose setae; article IV oval, shorter than article III, with twelve plumose setae; article V square to globular, with eight plumose setae; endite round, with one coupling hook and four circum-plumose setae laterally, with short bristles apically; epipod globular to oval, with fine setae on distal and mesial margin.

*Pereopods I–IV* (Fig. 10A–D) slender, without unguis, consecutively longer. *Pereopods V–VII* (Fig. 10E–G) consecutively shorter. *Pereopod I* (Fig. 10A), basis longest, with two plumose setae on distal end; ischium 0.3× as long as basis, with four plumose setae on inferior margin; merus globular to square, with numerous plumose setae inferiorly and two plumose setae on superodistal angle; carpus rectangular, with numerous plumose and serrated setae on inferior margin; propodus elongated oval, subequal in length to carpus, with numerous plumose setae on inferior margin, serrated setae on palmar surface and inferior margin, comb setae on distal end; dactylus small, with two comb setae, two plumose setae distally. *Pereopods II–IV* (Fig. 10B–D) similar to each other; basis to propodus with numerous plumose setae on inferior margin; merus to propodus with several simple setae; basis longer than ischium; merus similar to basis in length; carpus and merus subequal in length; propodus slightly shorter than carpus, with several simple setae on distal end; dactylus claw-like. *Pereopods V–VII* (Fig. 10E–G) resembling each other; basis with one or three penicillate setae on superior margin; ischium to carpus subequal in length; propodus with two penicillate setae on superior margin; dactylus bi-unguiculate, half of propodus.

*Pleopod I* (Fig. 11A), protopod rectangular, with three coupling hooks on inner margin; rami subequal, longer than protopod, with numerous plumose apical setae. *Pleopod II* (Fig. 11B) similar to pleopod I; protopod rectangular, with three coupling hooks on inner margin; rami subequal each other, wider than rami of pleopod I, longer than plumose setae, with numerous setae on distal end. *Pleopods III–V* (Fig. 11C–E) similar to each other; protopod short; endopod with rounded apex, with 0–2 plumose setae subapically; exopod gradually tapering distal end, almost 1.3× longer than endopod.

*Uropod* (Fig. 11F) elongated oval to rectangular; protopod ~ 3× longer than wide, with numerous fine setae on border; endopod oval to rectangular, with two plumose setae apically; exopod elongated triangular, with numerous fine setae on border.

##### Description of paratype male.

*Body* (Fig. 8D, E) cylindrical; length 3.3 mm; dorsal spines smaller than those of female. *Cephalon* (Fig. 8D, E) with two middorsal spines; anterolateral lobe rounded. *Pereonites* (Fig. 8D, E), pereonite I–III without dorsal spines; pereonite IV slender, narrower than that of female, with only one pair of dorsal spines posteriorly. *Pleopod I* (Fig. 11G), protopod rectangular, ~ 3.3× longer than wide, with four coupling hooks medially; rami similar in length, with plumose setae distally; exopod with fine setae subbasally; lateral notch of exopod present, but obscure. *Pleopod II* (Fig. 8F), protopod rectangular, with three coupling hooks on inner margin; plumose setae shorter than rami; endopod slightly longer than exopod, with seven plumose setae; exopod ~ 1.6× longer than protopod, with nine plumose setae; appendix masculine almost 1.8× longer than endopod, curved outwardly, acute distally.

##### Distribution.

Southern coast of South Korea.

##### Habitat.

Sublittoral zone of the rocky substrate.

##### Etymology.

The composite epithet of the specific name *trispinosus* is a combination of the Latin *tres*, meaning three, and *spinosus*, meaning thorny. This name refers to having three spines on the dorsal surface of the cephalon.

##### Remarks.

Within the genus, *Idarcturus trispinosus* sp. nov. is distinguished from *I. platysoma*, the type species of the genus, by having dorsal spines in both sexes (lacking the dorsal spines in *I. platysoma*) and the pleotelson bearing lateral wings (lacking in the latter) (Barnard 1914).

*Idarcturus trispinosus* sp. nov. is similar to *I. allelomorphus* in having dorsal spines, but the former differs by having three dorsal spines on the cephalon (vs. two in the latter) and six pairs of dorsal spines on pereonite IV (vs. one pair in the latter) (Menzies and Barnard 1959).

*Idarcturus trispinosus* sp. nov. most resembles *I. hedgpethi* in having anterolaterally expanded pereonite IV and dorsal spines. However, they could be distinguished by the anterolateral lobe of the cephalon (concave distally in the former vs. truncated in the latter) and the numbers of dorsal spines on the cephalon (three in the former vs. two in the latter), on pereonite IV (six pairs in the former vs. one pair in the latter), and on the pleon (two pairs in the former vs. one pair in the latter) (Menzies 1951).

#### Key to females of the species of *Idarcturus*

**Table:** 

1	Pereonites without dorsal spines	***I. platysoma* Barnard, 1914**
–	Pereonites with dorsal spines	**2**
2	Pereonites without spines dorsolaterally	***I. allelomorphus* Menzies & Barnard, 1959**
–	Pereonites with spines dorsolaterally	**3**
3	Cephalon with two middorsal spines; pereonite IV with one pair of dorsal spines; pleotelson with one pair of spines and two pairs of wings	***I. hedgpethi* Menzies, 1951**
–	Cephalon with three middorsal spines; pereonite IV with four pairs of dorsal spines; pleotelson with two pairs of spines and three pairs of wings	***I. trispinosus* sp. nov.**

#### Genus Neastacilla

Taxon classificationAnimaliaIsopodaArcturidae

Tattersall, 1921

38DD19B2-24BE-54DE-9497-423A1401589A

##### Type species.

*Astacilla falclandica* Ohlin, 1901, by subsequent designation.

##### Diagnosis

**(modified after King 2003b).** Body cylindrical; pereonite I fused to cephalon, occasionally indicated by groove dorsally or slit ventrally; pereonite IV ~ 3–10× longer than other pereonites; pereonites IV and V strongly geniculated. Antenna with one row of spines on each flagellar article. Pereopod I lacking an unguis; pereopods II–IV with claw-like dactylus, not flexible between carpus and propodus; pereopods V–VII with two claws on distal end of dactylus.

#### Neastacilla paralongipectus sp. nov.

Taxon classificationAnimaliaIsopodaArcturidae

D240BD0D-FC6D-5C54-AAF5-176C2F322CA8

http://zoobank.org/10892AFB-E914-4D4D-A466-DDFB880EA0A5

**Figure 12.**
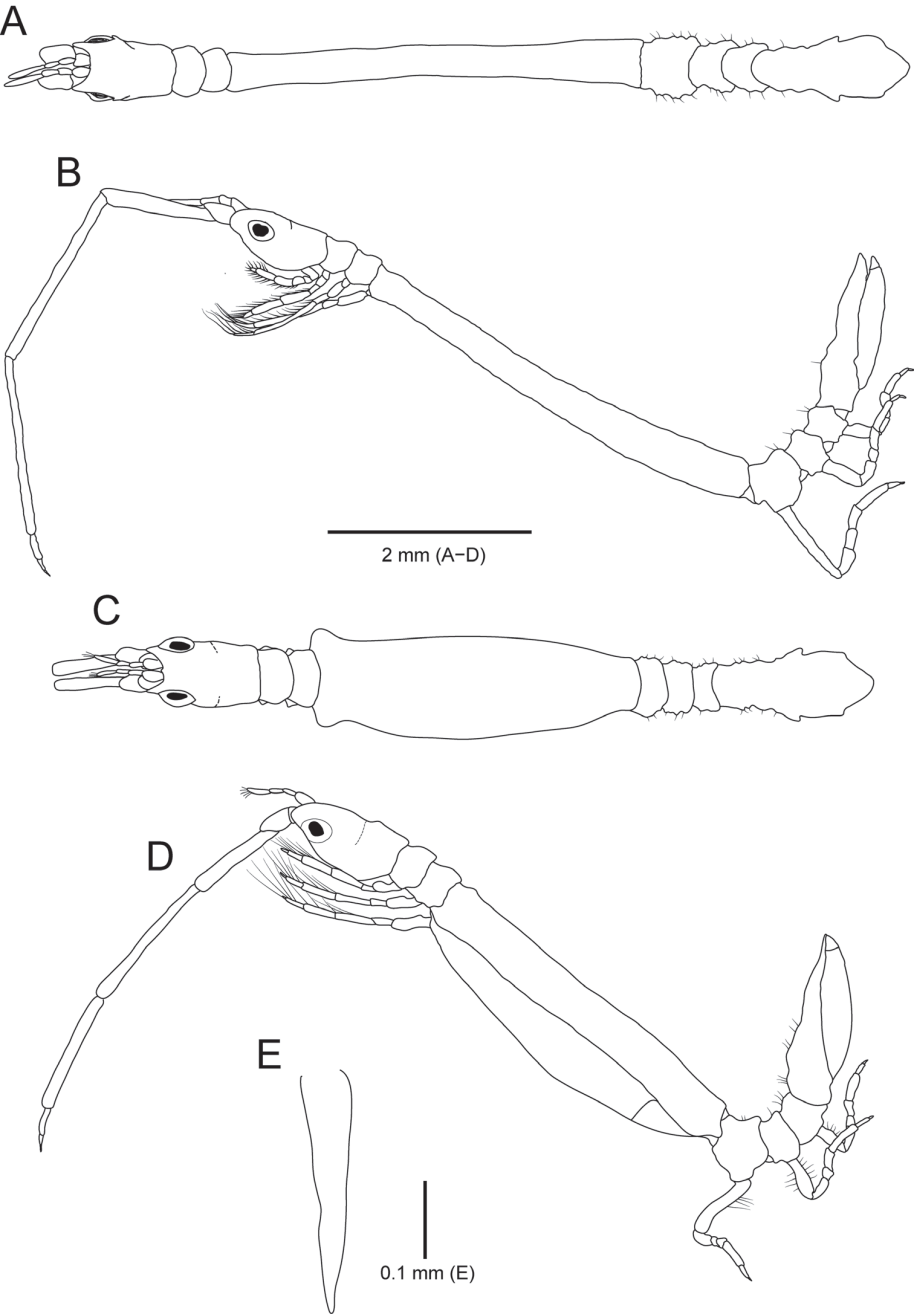
*Neastacilla paralongipectus* sp. nov., holotype, male **A** habitus, dorsal view **B** habitus, lateral view **E** penes. Paratype, female **C** habitus, dorsal view **D** habitus, lateral view.

**Figure 13.**
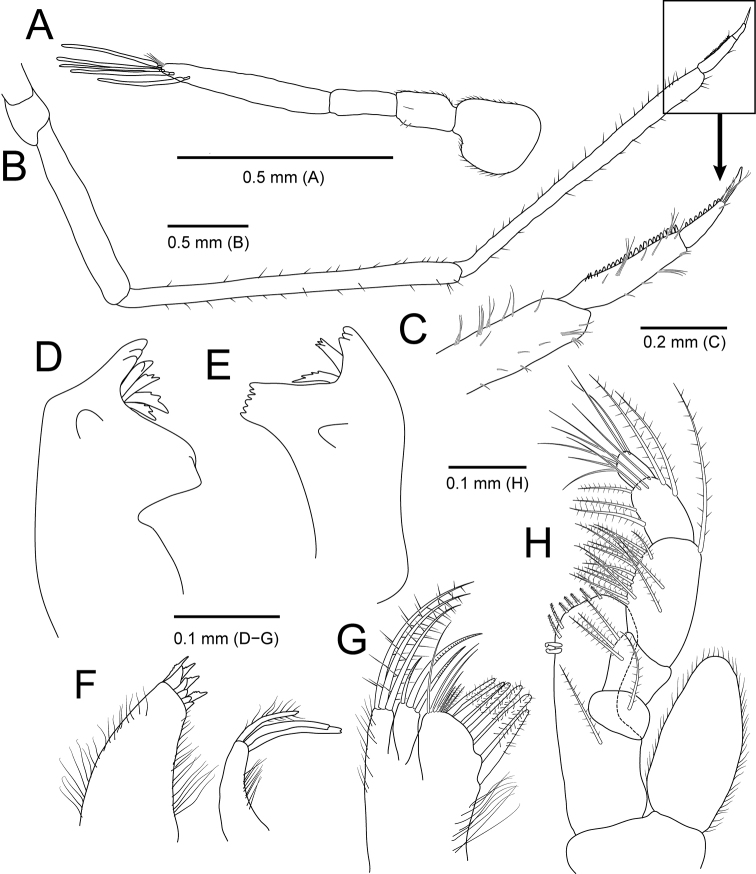
*Neastacilla paralongipectus* sp. nov., holotype, male **A** antennule **B** antenna **C** distal end of antenna **D** left mandible **E** right mandible **F** maxillule **G** maxilla **H** maxilliped.

**Figure 14.**
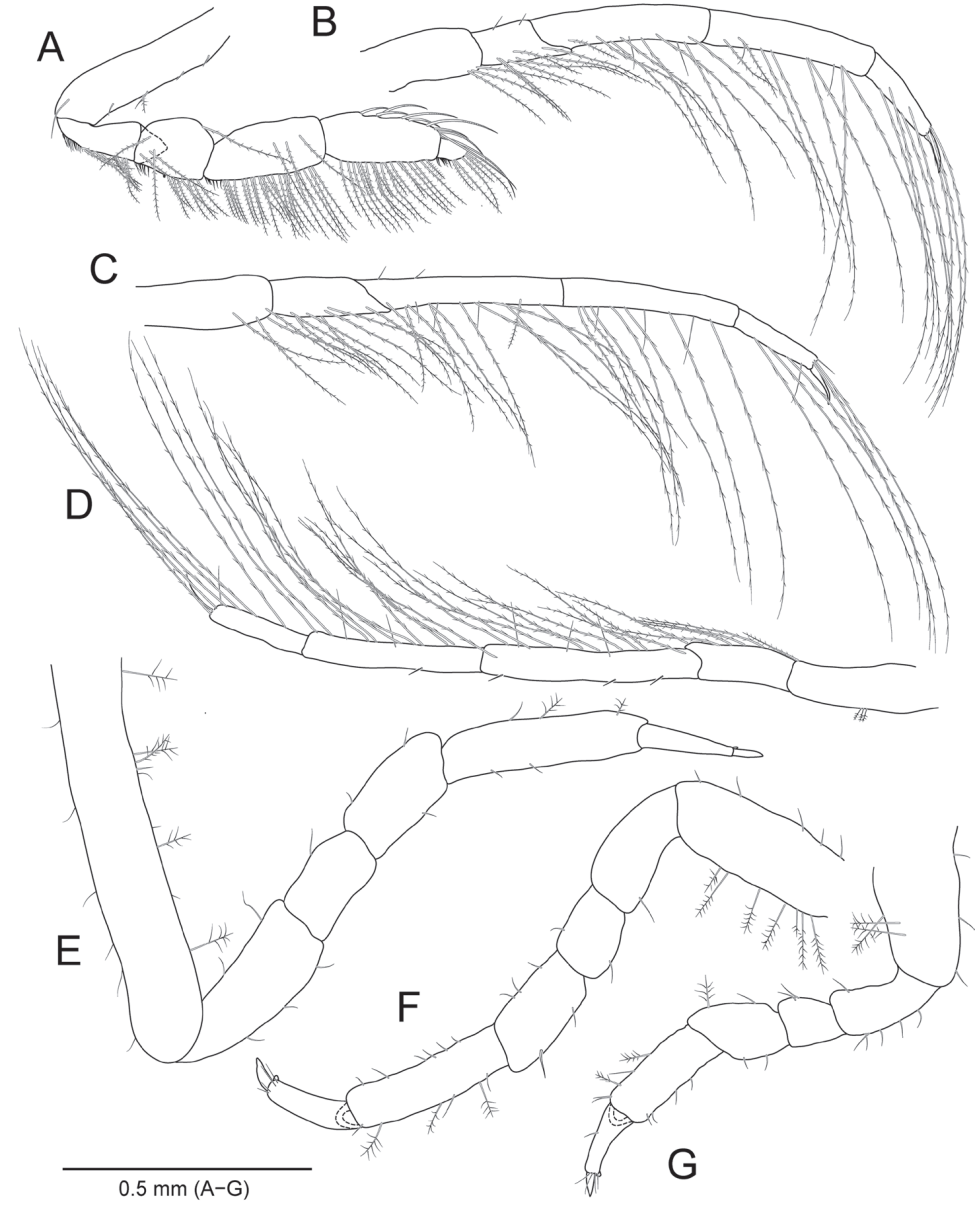
*Neastacilla paralongipectus* sp. nov., holotype, male **A** pereopod 1 **B** pereopod 2 **C** pereopod 3 **D** pereopod 4 **E** pereopod 5 **F** pereopod 6 **G** pereopod 7.

**Figure 15.**
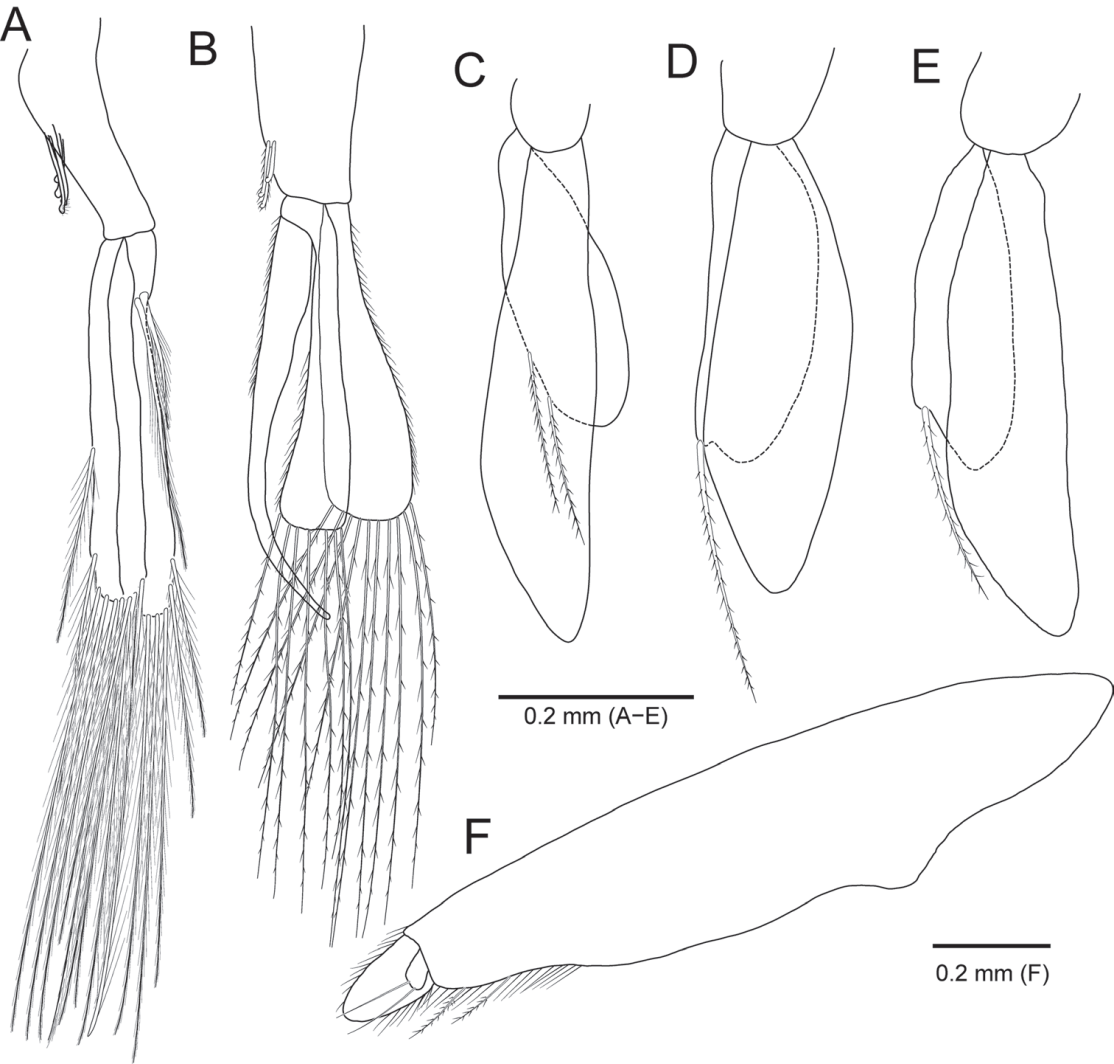
*Neastacilla paralongipectus* sp. nov., holotype, male **A** pleopod 1 **B** pleopod 2 **C** pleopod 3 **D** pleopod 4 **E** pleopod 5 **F** uropod.

Figures 12 , 13 , 14 , 15

##### Material examined.

***Holotype*.** South Korea • 1 ♂ (10.2 mm); Jeju-do, Seogwipo-si; 33°13'00"N, 126°19'30"E; 30 m; 31 Jan. 2018; Smith-McIntyre grab; NIBRIV0000862799.

***Paratypes*.** South Korea • 1 ovigerous ♀ (3.3 mm); Jeju-do, Seogwipo-si; 33°13'12"N, 126°32'12"E; 33 m; 1 Feb. 2018; Smith-McIntyre grab • 1 ovigerous ♀; Jeju-do, Seogwipo-si; 33°11'24"N, 126°18'18"E; 30 m; 28 Apr. 2018; Smith-McIntyre grab • 1 ♂; Jeju-do, Seogwipo-si; 33°13'54"N, 126°36'24"E; 38 m; 26 Apr. 2018; Smith-McIntyre grab; NIBRIV0000880422.

##### Description of holotype male.

*Body* (Fig. 12A, B) 10.2 mm, smooth, 12× as long as wide. *Cephalon* (Fig. 12A, B) with deeply concaved anterior margin possessing small median process; anterolateral lobe expended anteriorly; eye large, round, positioned on lateral margin. Pereonites II and III subequal in length; pereonite IV elongated, ~ 7× longer than pereonites II and III together; pereonites V–VII ~ 1.9× longer than pereonites II and III, surface of pereonites with small tuberculate elevations possessing setae. *Pleon* 1.3× longer than pereonites V–VII; pleotelson gradually tapering posterior region; apex rounded; lateral margin with two pairs of wings; proximal wing indistinct, but distal wing distinct.

*Antennule* (Fig. 13A) over second peduncular article of antenna, consisting of three peduncular articles and single-articled flagellum; peduncular article I square to globular, articles II and III cylindrical; article II slightly longer than article III; flagellum elongated oval, with five aesthetascs along with anterodistal region and three simple setae on distal end.

*Antenna* (Fig. 13B, C) slender, exceeding half of body length, composed of five peduncular articles and 3-articled flagellum; peduncular articles I and II subequal to each other; article IV longest; article V slightly shorter than article IV; articles IV and V with I–III pairs of simple setae; flagellar articles I and II with one row of spines resembling saw-teeth, article II with five simple setae on distal end.

*Left mandible* (Fig. 13D), incisor 3-toothed; lacinia mobilis 2-toothed, with three serrated setae; molar process broad, rough distally. *Right mandible* (Fig. 13E), incisor 3-toothed; lacinia mobilis 3-toothed, with one serrated seta; molar process, strongly serrated distally. *Maxillule* (Fig. 13F) with fine setae on lateral margin; inner lobe with one plumose seta and two distally bifid simple setae on distal end; outer lobe with four serrated robust setae and five robust simple setae on distal end. *Maxilla* (Fig. 13G) with fine setae laterally, consisting of three lobes; inner lobe with six stout circum-plumose setae, three plumose setae, four simple setae; mesial lobe with three comb setae; outer lobe with three plumose setae. *Maxilliped* (Fig. 13H), endite rounded distally, almost 1.2× wider than palp article III, with two coupling hooks medially, seven circum-plumose setae distally, one plumose seta mesially; palp article I with one plumose seta on mesial margin; article II with three plumose setae on medial margin; article III with 15 plumose on medial margin; article IV with five plumose setae medially and three simple setae distally; article V with six simple setae apically.

*Pereopods I–IV* (Fig. 14A–D) slender, sequentially longer, without unguis and flexion between carpus and propodus. *Pereopods V–VII* (Fig. 14E–G), sequentially shorter. *Pereopod I* (Fig. 14A) shorter than pereopods II–IV; basis longest, with one penicillate seta and four simple setae on superior margin, one simple seta on superodistal angle; ischium to dactylus with numerous plumose setae on inferior margin, 0–4 plumose setae on distal end; carpus and propodus subequal in length; propodus with ten comb setae on dorsal margin; dactylus as long as wide. *Pereopods II–IV* (Fig. 14B–D), basis to propodus with numerous plumose setae along with inferior margin; merus to propodus with several short simple setae on inferior margin; basis ~ 1.2× longer than ischium; ischium with oblique end distally; merus and carpus similar in length; dactylus reduced and claw-like. *Pereopods V–VII* (Fig. 14E–G) similar to each other; basis, with 3–7 penicillate setae on superior margin; ischium almost 1.7× longer than merus; merus and carpus subequal in length, with one penicillate seta on superior margin in pereopod VI; propodus with two or three penicillate setae on superior margin; dactylus bi-unguiculate, secondary unguis tiny.

*Penes* (Fig. 12E) simple, fused, with acute apex.

*Pleopod I* (Fig. 15A), protopod rectangular, with four coupling hooks medially; rami subequal, plumose setae longer than rami; exopod with six long plumose setae apically and two plumose setae subbasally, slightly notched subbasally; endopod with eight long plumose setae distally. *Pleopod II* (Fig. 15B) resembling pleopod I; protopod rectangular, ~ 0.8× shorter than protopod of pleopod I, with three coupling hooks on medial margin; exopod with ten long plumose setae on distal end, endopod with seven long plumose setae on apical end; appendix masculina 1.2× longer than endopod, tapering distally. *Pleopod III* (Fig. 15C), protopod square to globular; rami rounded distally; exopod 1.6× longer than endopod, without plumose setae, tapering distally; endopod with two plumose setae medially. *Pleopods IV and V* (Fig. 15D, E) resembling each other; protopod square to globular; rami with rounded distal margin; exopod almost 1.5× longer than endopod, without plumose setae; endopod with one plumose seta subapically.

*Uropod* (Fig. 15F) elongated oval; protopod ~ 4× longer than wide, with two plumose setae and several fine setae on subapical margin; exopod triangular, with numerous fine setae; endopod square to rectangular, with two simple setae on distal end.

##### Description of paratype female.

*Body* (Fig. 12C, D) ~ 10× as long as wide, length 7.5 mm. *Pereonite IV* (Fig. 12C, D) ~ 5.4× longer than pereonites II and III, shorter than male; anterolateral margins extended laterally. *Oostegite IV* (Fig. 12D) with suture line on posterior region.

##### Distribution.

Southern coast of Jeju-do in South Korea.

##### Habitats.

Sublittoral zone of sandy bottom.

##### Etymology.

The composite epithet of the specific name *paralongipectus* is a combination of the Greek prefix *para*- and the specific name of Neastacilla longipectus Nunomura, 2008, which means near longipectus, refering to the close resemblance to *N. longipectus*.

##### Remarks.

Seven species of *Neastacilla* have pereonite IV at least 5× as long as pereonites II and III together in female as in the new species: *N. algensis* Hale, 1924, *N. deducta* Hale, 1925, *N. kanowna* King, 2003, *N. lawadi* King, 2003, *N. longipectus* Nunomura, 2008, *N. monoseta* (Guiler, 1949), and *N. soelae* King, 2003 (Hale 1924, 1946; King 2003b; Nunomura 2008). Among these species, *Neastacilla paralongipectus* sp. nov. is easily distinguishable from *N. algensis*, *N. lawadi*, and *N. soelae* in terms of the absence of dorsal tubercles on the cephalon in female (vs. presence in the latter species) (Hale 1924; King 2003b). *Neastacilla paralongipectus* sp. nov. is similar to *N. deducta*, *N. kanowna*, and *N. monoseta* in having a smooth body lacking dorsal elevations (King 2003b). However, *Neastacilla paralongipectus* sp. nov. differs from *N. deducta*, *N. kanowna*, and *N. monoseta* in that oostegite IV has suture posteriorly (vs. mesially in *N. deducta* and *N. monoseta*) and the pleotelson has rounded apex (vs. truncated in *N. kanowna*) (King 2003b). *Neastacilla paralongipectus* sp. nov. is easily distinguishable from *N. longipectus* in terms of the shape of the anterior margin of the cephalon (deeply concave in the former vs. slightly concave in the latter), the shape of the penes (elongated triangle in the former vs. rectangle in the later), and the structure of pereonite I and pleonite I (fused to cephalon and pleotelson, respectively, in the former vs. not fused in the latter) (Nunomura 2008).

#### Key to known species of the genus *Neastacilla* in the northwest Pacific

**Table:** 

1	Eye absent	**2**
–	Eye present	**4**
2	Body with bosses	***N. ochroleuca* Kussakin & Vasina, 1990**
–	Body without bosses	**3**
3	Carpus 3.2× longer than wide in pereopod	***N. birsteini* Golovan, Malyutina & Brandt, 2018**
–	Carpus ~ 6× longer than wide in pereopod I	***N. anophthalma* (Birstein, 1963)**
4	Pereonite IV ~ 0.5–3× longer than pereonites II and III together	**5**
–	Pereonite IV at least 7× longer than pereonites II and III together	**15**
5	Pleotelson without lateral wings	**6**
–	Pleotelson with lateral wings	**8**
6	Pereonite IV similar with other pereonites in length	***N. tritaeniata* (Richardson, 1909)**
–	Pereonite IV ~ 2× longer than pereonites II and III together	**7**
7	Eye small and without pigment	***N. leucophthalma* Kussakin, 1971**
–	Eye large and black	***N. tzvetkowae* Kussakin, 1974**
8	Body covered by tubercles	**9**
–	Body covered by spines	**11**
–	Body smooth	**14**
9	Tubercles small and granule size	***N. scabra* Nunomura, 2006**
–	Tubercles large and prominent	**10**
10	Eye small and without pigment	***N. nodulosa* Kussakin, 1982**
–	Eye large and with pigment	***N. pallidocula* Kussakin & Vasina, 1990**
11	Spines large and prominent	**12**
–	Spines minute and granule size	**13**
12	Pereonite IV 1.5× longer than pereonites II and III together	***N. exilis* Kussakin, 1971**
–	Pereonite IV similar to pereonites II and III together	***N. spinifera* Nunomura, 2006**
13	Pleotelson with rounded apex	***N. littoralis* Kussakin, 1974**
–	Pleotelson with acute apex	***N. richardsonae* Kussakin, 1982**
14	Lateral wings of pleotelson small and indistinct	***N. kurilensis* Kussakin, 1974**
–	Lateral wings of pleotelson large and distinct	***N. polita* ( Gurjanova, 1936)**
15	Pleotelson without lateral wings	***N. tanakai* Nunomura, 2004**
–	Pleotelson with lateral wings	**15**
16	Pereonite I separated from cephalon; pleon 2-segmented; anterior margin of cephalon deeply concave	***N. longipectus* Nunomura, 2008**
–	Pereonite I fused to cephalon; pleon single-segmented; anterior margin of cephalon slightly concave	***N. paralongipectus* sp. nov.**

## Supplementary Material

XML Treatment for Amakusanthura intermedia

XML Treatment for Apanthura laevipedata

XML Treatment for Arcturidae

XML Treatment for Idarcturus

XML Treatment for Idarcturus trispinosus

XML Treatment for Neastacilla

XML Treatment for Neastacilla paralongipectus
